# A correct diagnosis and close, high-quality follow-up care are crucial for successful management of myasthenia gravis patients

**DOI:** 10.1055/s-0046-1825753

**Published:** 2026-11-03

**Authors:** Carla A. Scorza, Fulvio A. Scorza, Josef Finsterer

**Affiliations:** 1 Universidade Federal de São Paulo, Escola Paulista de Medicina, São Paulo SP, Brazil.; 2 Neurology & Neurophysiology Center, Department of Neurology, Vienna, Austria.

Dear Editor,

Brazilian patients with ocular myasthenia or myasthenia gravis (MG) collected over a 14-year period through the Brazilian public health system (SUS, from the Portuguese Sistema Único de Saúde). 1 It was found that the number of hospital admissions due to MG increased during the observation period, that 31% of patients suffered exacerbations, and that 9% suffered crises. 1 It was concluded that the prevalence of MG increased during the observation period, which complicates the treatment of these patients. 1 The study is interesting, but some points should be discussed.

The first point is that no distinction was made between MG based on antibodies against the acetylcholine receptor (AChR), against muscle-specific kinase (MUSK), or against the low-density lipoprotein receptor-related protein (LRP4). The distinction between these forms is crucial, as the treatment and course of these different types of MG vary greatly. 2 It is also unclear whether patients with myasthenic syndrome were included in the study or not.

The second point is the increasing crisis rate over the years. 1 One would expect treatment to become more efficient and targeted over the years as experience with this disease increases, but the opposite has been the case. What is the reason for the increase in crises? Was this due to declining patient compliance, the inefficiency of the immunosuppressive therapy used, or were there specific triggers for increased antibody production? A distinction should also be made between myasthenic and cholinergic crises, as the treatment of both types varies considerably.

The third point is that the study did not take into account the number of patients who underwent thymectomy. 1 Since MG is associated with thymoma in 10 to 15% of cases 3 and patients under the age of 60 can benefit from thymectomy, 4 it is crucial to know how many had thymoma and how often thymectomy was performed to treat MG.

The fourth point is that the diagnostic criteria used to diagnose MG were not listed in the methodology sections. Of particular interest is how many of the included patients had seronegative MG and to what extent seronegative patients contributed to the frequency of treatment failures. It is also important to know how many patients had their MG diagnosis revised over time.

The fifth point is that no distinction was made between patients with ocular myasthenia and patients with generaslised MG. How many patients with ocular myasthenia developed MG and how many had MG completely resolved over time?

The sixth point is that the quality of follow-up care was not reported. 1 How often were patients invited for follow-up care, how often were they tested for antibody titers, subjected to repeated nerve stimulation, or clinically assessed? How many of the patients experienced side effects from azathioprine or rituximab?

In summary, correct diagnosis and close, high-quality follow-up care are crucial for the satisfactory treatment of MG patients.
